# The Perception of Dynamic and Static Facial Expressions of Happiness and Disgust Investigated by ERPs and fMRI Constrained Source Analysis

**DOI:** 10.1371/journal.pone.0066997

**Published:** 2013-06-20

**Authors:** Sina Alexa Trautmann-Lengsfeld, Judith Domínguez-Borràs, Carles Escera, Manfred Herrmann, Thorsten Fehr

**Affiliations:** 1 Center for Cognitive Sciences (ZKW), Department of Neuropsychology and Behavioral Neurobiology, Bremen University, Bremen, Germany; 2 Department of Neurophysiology and Pathophysiology, University Medical Center Hamburg-Eppendorf, Hamburg, Germany; 3 Cognitive Neuroscience Research Group, Department of Psychiatry and Clinical Psychobiology, University of Barcelona, Barcelona, Catalonia, Spain; 4 Laboratory for Behavioral Neurology and Imaging of Cognition, Department of Neuroscience, University Medical Center, Geneva, Switzerland; 5 Hanse-Wissenschaftskolleg, Delmenhorst, Germany; 6 Institute for Brain, Cognition and Behavior (IR3C), University of Barcelona, Barcelona, Catalonia, Spain; 7 Center for Advanced Imaging, Bremen University, Bremen, Germany; 8 Department of Neurologie II, Otto-von-Guericke-University Magdeburg, Magdeburg, Germany; Max Planck Institute for Human Cognitive and Brain Sciences, Germany

## Abstract

A recent functional magnetic resonance imaging (fMRI) study by our group demonstrated that dynamic emotional faces are more accurately recognized and evoked more widespread patterns of hemodynamic brain responses than static emotional faces. Based on this experimental design, the present study aimed at investigating the spatio-temporal processing of static and dynamic emotional facial expressions in 19 healthy women by means of multi-channel electroencephalography (EEG), event-related potentials (ERP) and fMRI-constrained regional source analyses. ERP analysis showed an increased amplitude of the LPP (late posterior positivity) over centro-parietal regions for static facial expressions of disgust compared to neutral faces. In addition, the LPP was more widespread and temporally prolonged for dynamic compared to static faces of disgust and happiness. fMRI constrained source analysis on static emotional face stimuli indicated the spatio-temporal modulation of predominantly posterior regional brain activation related to the visual processing stream for both emotional valences when compared to the neutral condition in the fusiform gyrus. The spatio-temporal processing of dynamic stimuli yielded enhanced source activity for emotional compared to neutral conditions in temporal (e.g., fusiform gyrus), and frontal regions (e.g., ventromedial prefrontal cortex, medial and inferior frontal cortex) in early and again in later time windows. The present data support the view that dynamic facial displays trigger more information reflected in complex neural networks, in particular because of their changing features potentially triggering sustained activation related to a continuing evaluation of those faces. A combined fMRI and EEG approach thus provides an advanced insight to the spatio-temporal characteristics of emotional face processing, by also revealing additional neural generators, not identifiable by the only use of an fMRI approach.

## Introduction

Numerous studies have examined emotional face processing with functional imaging methods and revealed a network of brain regions involved in the processing of emotional facial expressions, including the amygdala, insula, superior and medial temporal, and inferior frontal regions with a high spatial resolution (for review, see [1]). To study emotion perception with a high temporal resolution, the perception of static emotional facial expressions (for review, see [2]; [3]) has been investigated with event-related potentials (ERPs).

### ERP Components due to Emotional Facial Expression Processing

Three prominent ERP-components are hypothesized to be modulated by emotional content during static emotional face processing: (1) Emotional face perception studies in monkeys [4] and scalp and intracranial recordings in humans have claimed that the N170 is face- but not emotion-specific [2], [5]. In addition, a double dissociation has been reported in human patients suffering from prosopagnosia due to brain lesions in either inferior occipito-temporal areas or right occipital and parietal areas. These patients were unable to recognize faces, but they were able to distinguish different emotional expressions. Furthermore, they have shown non-selective or absent N170 during the presentation of faces supporting the face specificity of the N170 [2]. However, several studies in human individuals found strong evidence for an emotional modulation of the N170 [6]–[8]. The reason for these contradictory findings could be due to different experimental designs. While the latter studies not finding an emotional modulation of the N170, applied explicit categorization tasks, the former studies, reporting an emotional modulation of the N170, applied passive or attentive viewing tasks without explicit categoriozation of emotional stimuli. Therefore, it seems that the emotional modulation of the N170 rather depends on specifications of the experimental setup, and it seems to be less dependent on the neural processing of facial stimuli per se.

(2) The EPN (early posterior negativity) reflects a relatively negative shift at posterior-lateral electrodes between 250 and 350 ms. In previous studies, disgust [9] and threat perception [10] enhanced the amplitude of the EPN. This negative deflection has been associated with the allocation of attention and sensory resources -also labeled “tagging” [10]- of motivationally relevant, predominantly negatively valenced stimuli, for facilitating further processing of emotions. Other studies reported an enhanced EPN for both positive and negative emotional stimuli [11], [12].

(3) The LPP (late positive potential) component has been shown to be most prominent over midline centro-parietal regions during the attentive processing of both positive and negative emotional facial expressions. This finding was discussed in relation to a continued and deeper evaluation of emotional stimuli [3], [11].

### Emotional Facial Expression Processing and fMRI-constrained Source Approaches

In addition to ERP analysis, source analysis provides insight into possible underlying generators of ERP activations. To date, there have been several EEG [6], [8], [13], [14] and magnetoencephalography (MEG) studies [15]–[19] applying source localization procedures. Some of these studies investigated the temporo-spatial dynamics of topographically different brain activations during emotion perception of either facial expressions [6], [14], [18], [19], or emotionally pleasant or unpleasant stimuli [20], [21]. Other studies investigated temporo-spatial dynamics of topographically different brain activations during a perceptual matching task of facial emotional expressions investigated with MEG [15]. A limited number of studies used knowledge from fMRI blood-oxygen-level-dependent (BOLD) analyses to arrange appropriate EEG source models, refered to as so-called fMRI constrained source analysis approach [22]. Constraints have been shown to improve the validity of source models, reflected in higher rates of common variance explanation [23]–[25]. Moreover, they enhance the accuracy of seeded source models because spurious sources can largely be ruled out (see [26], for a more detailed methodological discussion of mismatched sources in EEG and fMRI).

Source models for emotional face perception designs by, for example, Lewis and co-workers in a previous MEG study [18] and Sprengelmeyer and Jentzsch [14] in a previous EEG study indicated source activation in the fusiform gyrus and adjacent visual areas either unilaterally or symmetrically, whereas other MEG studies reported amygdala activation by applying beamforming approach [15], [17].

One study on emotion processing of IAPS (International Affective Picture System) pictures [22] applied an fMRI constrained source analysis approach focusing solely on the LPP time window between 400 and 900 ms after stimulus onset. The authors showed that, within this time window, emotional stimuli produced a peak activation in lateral occipital, inferior temporal, and medial parietal cortex in comparison to neutral stimuli. Source moments showed high correlations with fMRI BOLD signal in the respective regions. In the present study, spatial BOLD-fMRI data from a prior study [27] applying the exact same experimental setup were used to improve the validity of respective combined seeded and extended source models, and to provide more insight into the spatio-temporal dynamics of perceptual emotional facial expression processing of realistic dynamic stimuli. Additionally, as mismatches between BOLD activation foci and EEG-sources have previously been reported [26], spatial information yielded in the prior fMRI-study by Trautmann et al. [27] were complemented with additionally fitted sources.

### Potential Enhancement of Ecological Validity by the use of Realistic Dynamic Stimuli

Most of the studies on emotional face perception have used static faces as stimuli. Behavioral studies have demonstrated that dynamic stimuli can be more accurately recognized than the static ones by healthy, autistic, and mentally retarded humans [28]–[30]. There is only scarce information on the spatio-temporal differences between the neural processing dynamics of static and dynamic emotional stimuli. In this sense, neuroimaging studies provided evidence that dynamic emotional expressions evoke more widespread activation patterns in emotion-related brain regions compared to static emotional ones with high spatial precision, but lacking precise temporal information [27], [31], [32]. An ERP study by Recio and co-workers [12] investigated the differences of static and dynamic emotional face processing of anger, happiness, and neutral expression in an explicit face categorization task. They reported, among other effects, an emotionally modulated LPP enhancement for dynamic compared to static facial expressions over posterior-central regions. Other studies examined the time-course of dynamic emotional face processing by using ERPs and a gaze direction cueing task with dynamic emotional faces and eye gaze [33], or by applying static and dynamic facial expressions in a steady state design [34]. A crucial problem of different study designs involving cognitive task elements is, however, that putatively minor changes in the experimental setup can produce fundamentally different neural responses [35]. Therefore, the experimental setup of the prior approach was not changed in the current study.

### Aims and Hypotheses of the Present Study

The present study aimed at investigating the spatio-temporal-dynamics of emotional facial expression processing of static and dynamic stimuli in an attentive viewing task, by means of ERPs, ERP-topographies, and discrete fMRI constrained source analyses, complemented with additionally fitted sources. The exact same study protocol as reported in a previous fMRI study by Trautmann et al. [27] was applied, and the reported fMRI data served as constraints and a priori knowledge for the present source analyses.

Three main hypotheses and assumptions were tested: (1) Better recognition accuracy of different emotional categories of facial expressions and higher arousal rates for dynamic compared to static emotional facial expressions, as measured by an explicit rating task after the EEG recordings, were expected [9], [10]. (2) For the static stimulus condition, enhanced mean amplitudes of the N170 were predicted for emotional compared to neutral stimulus processing [6]–[8] because we applied an attentive viewing task without explicit emotional categorization of our emotional stimuli. In addition, we expected enhanced amplitude of the LPP component for static facial expressions [11], [36]. Furthermore, enhanced EPN amplitudes for static emotional compared to neutral stimuli were expected [9]–[12]. (3) We predicted enhanced LPP for both dynamic emotional stimuli compared to neutral stimuli based on a previous study by Recio and colleagues [12]. More specifically, for the dynamic stimulus presentation condition, LPP amplitudes for emotional compared to neutral stimuli were predicted to be enhanced over longer time epochs and over more widespread electrode sites based on data presented in recent imaging studies [27], [31], [32].

Additionally, spatio-temporal dynamics of emotional facial processing were further investigated in detail in a source analysis approach: Based on our previous fMRI study (Trautmann et al., 2009), we expected that in the dynamic stimulus modality sources of a more widespread network of generators differentially contribute to the explanation of the respective seeded source model. Furthermore, we predicted that sources representing posterior regions would be activated earlier in relation to early perceptual processing steps than sources in frontal brain regions expected to be related to later conceptual and/or appraisal-related processing of the respective emotional stimuli.

In particular for static stimuli, we expected to potentially fit sources in posterior regions in addition to the seeded source model because only frontal regions have been shown to be activated in our prior fMRI study [27]. The reason for finding only posterior activations for static stimuli was probably due to the fact that some sources had been invisible to an fMRI approach. In fact, underlying neural processing steps in neural sub-networks can be of transient and very fast nature. Hence, fMRI might have not detected the generators in posterior regions because the fMRI approach is rather related to brain activations integrated over time because of its low temporal resolution (for a more detailed discussion of these results, please refer to [26], [27]).

## Methods

### Study Participants

Nineteen female university students (mean age 21.3±3.0 (SD) years, range: 13–20 years, range 18–28 years; education: 14.6±2.3 years) with normal or corrected to normal visual acuity, no history of neurological or psychiatric illness, no drug abuse, no current psychotropic medication and right handedness according to the Edinburgh Handedness Inventory Questionnaire [37] met the inclusion criteria of the study and gave informed and written consent to participate in the present study. The study protocol was approved by the local ethics committee of the University of Barcelona and was designed according to the Code of Ethics of the World Medical Association (Declaration of Helsinki, 1964). Participants were naïve to both the working hypotheses of the study and the stimulus material. The reason for choosing only women concerns homogeneity of the sample, as we did in previous fMRI studies [27], [38]. Furthermore, we intended to achieve homogeneity with regards to gender between our fMRI study and the present study- given the well-known gender differences in emotional processing [39], [40], or the structural dimorphism between men and women in limbic regions [41]. Furthermore, as previously reported, women showed more widespread activations of emotion-related areas in response to emotional stimuli [42].

### Experimental Design and Stimuli

**Figure 1:**
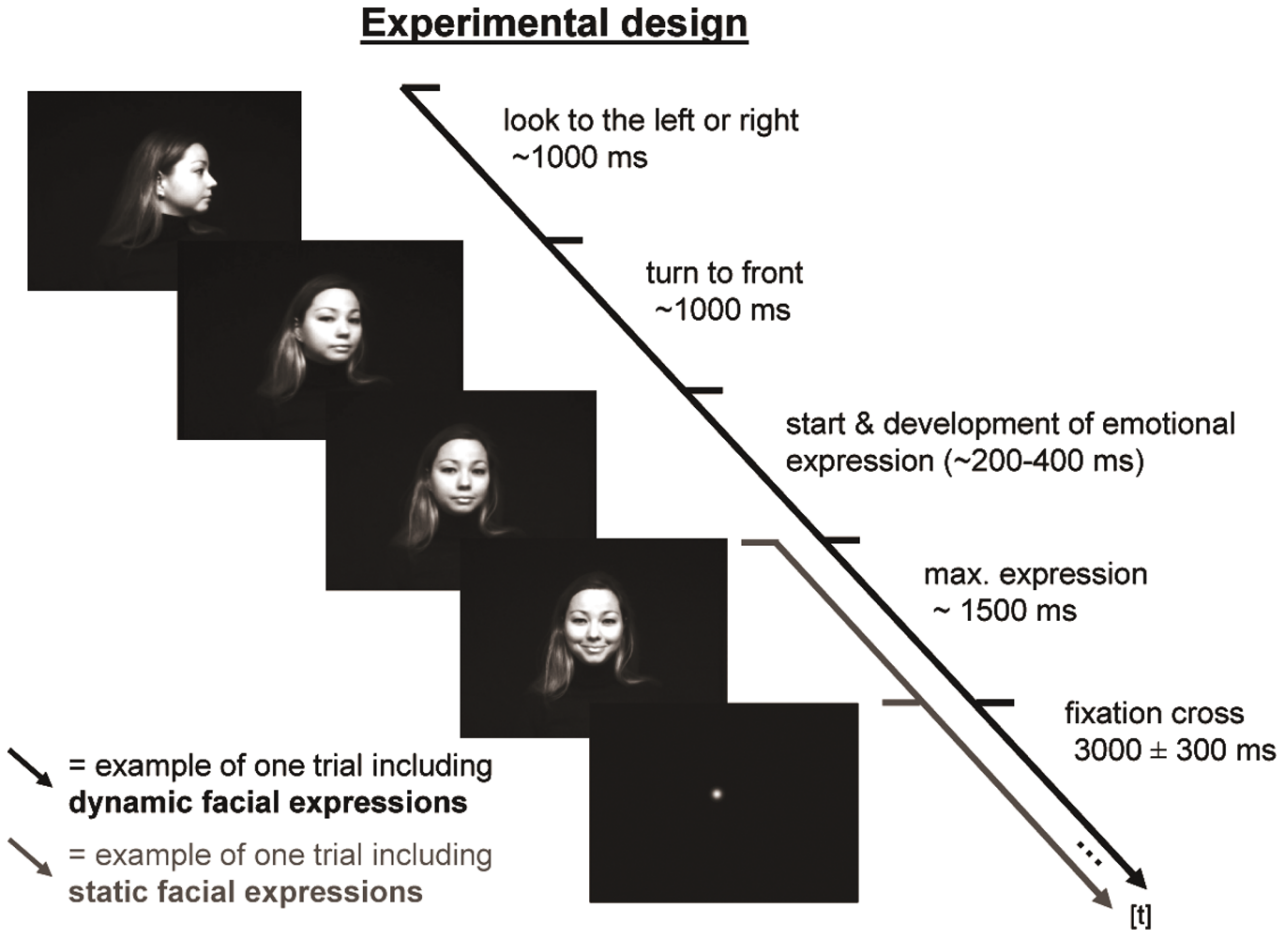
Experimental design. One exemplary trial is depicted including presentation durations in ms of a dynamic and static happy facial expression indicated by a black and grey arrow, respectively. Please note that the subject of the photograph has given written informed consent, as outlined in the PLOS consent form, to publication of her photograph.

We applied the same experimental design as reported in a previous fMRI study [27]. Dynamic and static facial expressions (40 stimuli per emotional category [neutral, happiness, disgust] and per modality [static, dynamic]) were presented in a pseudo-randomized non-stationary probabilistic sequence [43] and were counterbalanced in two separate runs across participants. Each run consisted of four separate phases, each including 30 stimuli of different emotional category interleaved with short breaks to avoid fatigue in participants. Facial expressions were taken from 40 different female actresses. Each actress was presented once showing each a neutral, happy, and disgusted facial expression both in the static and dynamic modality. Dynamic stimuli had an average duration of 3.7 seconds. At the beginning of the video stimuli the profiles of the displayed actresses showed a neutral expression presented from the right or left side for 1 sec. The actresses then turned to the front, and started an emotional expression (happiness, disgust), which dynamically developed to its maximum intensity. This maximum expression intensity was displayed for 1500 ms on average until the end of the video (see Fig. 1 for illustration). Analogously, static stimuli, only displaying the maximum facial expression, were as well presented for 1500 ms. Dynamic and static stimuli were followed by a fixation dot for 3000±300 ms jittered. For a more detailed description of the creation and validation of the stimulus data base please refer to Trautmann and collegues [27].

Participants were asked to watch the stimuli attentively on a screen (distance 120 cm, size H 15×W 18.33 cm, vertical and horizontal visual angle of 7.1° and 8.7°, respectively) and to “empathize” with the displayed facial expressions. After the EEG recording session, participants evaluated each individual stimulus presented in the same sequence as during the EEG session according to two scales: Arousal (0 = not to 10 = highly arousing), and emotional stimulus category (neutral, disgust and happiness). For more detailed information about previous evaluation of the stimulus material in healthy German participants please refer to Trautmann and colleagues [27].

### Behavioral Data Analyses

Behavioral data were analyzed according to the mean arousal rates and mean accuracy rates for each emotional category rating (happiness, disgust, neutrality). Repeated measurement ANOVAs (calculated with SPSS© Inc., Chicago, USA) were calculated for emotional CATEGORY (CAT, three levels: Neutrality, happiness, disgust)×stimulus presentation MODALITY (MOD, two levels: Dynamic, static). In case of significant or trend to significant interaction effects, paired sample t-tests were calculated for post-hoc comparisons.

### EEG Procedures and Analyses

Multi-channel EEG was recorded in an electrically and sound shielded room from 62 Ag/AgCl scalp electrodes placed according to the international 10-10-system (average reference, A/D-rate 512 Hz, Eemagine from ANT, B.V., Enschede, Netherlands) including horizontal and vertical electro-oculogram attached to the right canthus and below the right eye, and ground electrode placed on the chest of each participant. Impedances were kept below 15 kOhm and were checked repeatedly between runs.

EEG data were analyzed with BESA® 5.1.8.10 (www.besa.de, MEGIS Software, Munich, Germany) for ERP averaging and with Matlab® Tools (version 6.5.1, MathWorks Inc.; Aachen, Germany) to calculate mean amplitude values.

Data of each participant were visually inspected for artifacts and slow drifts. Channels including drifts were corrected by the spherical spline interpolation algorithm implemented in BESA® Software before averaging. The number of interpolated channels was kept below 5% (Picton et al. 2000) of the complete channel set up (maximally excluded channels: three, equals max. 4.7%).

For stimulus-locked analyses, visual inspection of each dataset indicated that the data presented a low eye-blink-rate, saccade-, and muscle-artifacts. Trials with artifacts showing amplitudes larger than 100µV were excluded from further analyses by applying the artifact scanning tool provided by BESA®-Software. Artifact-free ERP data included an average of 33.3 sweeps for static (neutrality 33.1±4.3, happiness 33.7±4.5, disgust 33.1±5.0) and 34.9 sweeps for dynamic (neutrality 35.3±2.5, happiness 34.1±2.6, disgust 35.2±2.5) stimulus categories.

Data were high-pass filtered (0.1 Hz, 6 db/octave, forward) and averaged over trials from −100 ms (baseline) to 1000 ms locked to stimulus onset for static faces. For dynamic faces, data were averaged over trials from −100 ms to 1000 ms locked to the beginning of the emotional expression determined by an independent visual frame-by-frame inspection of three raters, i.e. after the actor looked to the right/left for one second, turned to the front displaying a neutral face and then started the emotional expression. The first author of the present study and two further independent raters (see also Trautmann et al. 2009 for details) identified the video frame latency of the beginning (zero-point for stimulus-locked ERPs) and the maximum of the respective emotional facial expressions. The facial expression developed to its maximum expression within approximately 200–400 ms and remained at the maximum to the end of the video. Hence, the presence of early and strictly time-locked components in the dynamic condition, i.e., N170, was only anticipated during the onset of the dynamic stimulus displays when individuals showed a neutral facial expression looking to the left/right showing their profile. Exploratory ERP analysis time-locked to the beginning of each video separately for each emotional condition revealed no significant amplitude differences for early ERP-components. This was expected because of the neutrality of the facial expressions at the beginning of all videos. Thus, when emotional expressions actually began after faces have turned to the front with neutral expressions, the emotional expression develops and remains on the screen for a mean duration of 1500 ms. Hence, the video consisted of a continuous stream of facial expression information, and therefore, no early components for emotional dynamic expressions were expected. However later components reflecting sustained activation such as, for example, the LPP were expected.

For both the static and the dynamic presentation modalities, a low-pass filter (30 Hz) was applied to individual ERPs before calculating grand averages. Time windows for further analyses were determined based on both previous findings and identifiable deflections for static faces. The following mean amplitudes were included in the statistical analysis: 140–190 ms (N170), 250–350 ms (EPN), and 600–800 ms (LPP). For dynamic stimuli, seven 100 ms time windows (100–800 ms) were determined as ERPs indicated sustained activity during this time range.

**Figure 2:**
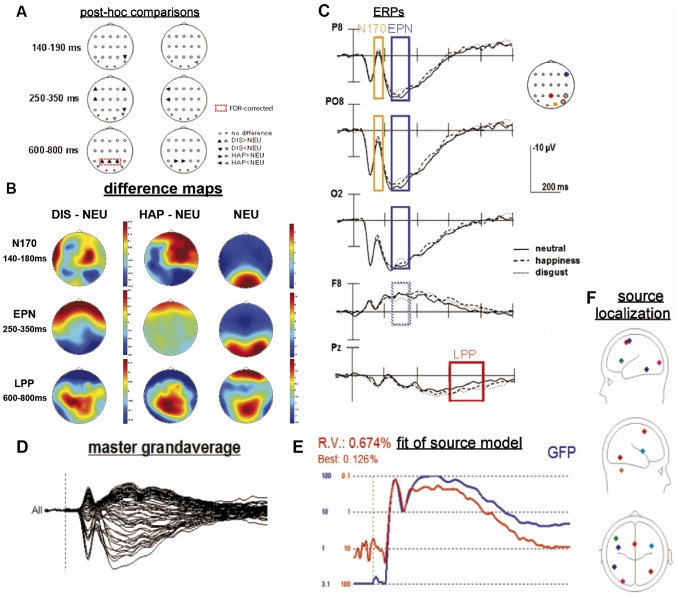
Results of ERP and fMRI constrained source analysis of static facial expression. (A) posthoc comparisons for three selected time windows (dependent t-tests, p<.05). Dashed red boxes represent significant results after FDR-correction. (B) Topographical maps of difference waves for disgust (DIS)>neutral (NEU) and happy (HAP)>NEU and for neutral alone. (C) ERPs for selected posterio-lateral, -midline and anterio-lateral electrode positions. Orange, blue and red boxes denote the analyzed time windows (fN170, EPN, LPP, respectively) including its significant category effects. Orange, blue and red dots in the head model in the upper right corner represent the displayed electrodes of interest and the corresponding time windows. Source model of static stimuli: (D) master grand-average over all electrodes and all conditions (−100–1000 ms), (E) fit of source model indicated by global field power curve (GFP; blue curve) and explained variance of the model (residual variance [RV] and best fit; red curve), (F) Eight RS (each displayed in individual color) projected onto a 4-shell spherical model displayed from 3 different perspectives (saggital [left, right], transversal [above]. Note: RS one to four were seeded based on fMRI activations and RSs five to eight were added by sequential fitting procedure.

According to the above-determined time windows, mean amplitudes were calculated prior to statistical analyses of the data. For topographical ERP analyses, repeated measurement ANOVAs, including 15 approximately equidistant distributed EEG channels (F7, F3, Fz, F4, F8, T7, C3, Cz, C4, T8, P7, P3, Pz, P4, P8) and encompassing within-subject factors ANTERIOR-POSTERIOR (AP, three levels: Frontal, central and posterior electrode positions), LATERALITY (LAT, five levels: From right to left electrode sites), and CATEGORY (CAT, three levels: Neutral, happiness, and disgust), were calculated separately for static and dynamic modalities. Further additional electrodes of interest (AF7, AF8, F5, F6, CP3, CP1, CPz, CP2, CP4, PO7, PO3, POz, PO4, PO8, O1, O2) were analysed with ANOVAs with the factor CAT (see also Fig. 2A/3A, filled grey circles).

In case of significant or trend to significant interaction effects including the factor CAT, paired sample t-tests were calculated for post-hoc comparisons without correction for multiple comparisons. We furthermore report FDR-corrected post-hoc comparisons for all conducted EEG analyses over all electrode sites including equidistant sensors and additional electrodes of interest for each time window (N170, EPN, LPP for static modality and for the remaining six significant 100 ms time windows from 200–800 ms for dynamic modality). Thus 93 comparisons were included in the calculation of FDR-corrected p-values for each time window.

### FMRI Constrained Source Analysis

Because of potentially mismatching generators in fMRI and EEG (for a detailed and critical discussion, see [26]), we seeded an fMRI-constrained source model, based on the results observed by Trautmann et al. [27], complemented with additionally fitted regional sources (RSs) for the following reasons:

First, constraints improve the explanatory value of the source model applied to ERP-data, especially when based on *a priori* knowledge [23]–[25]. One of the shortcomings of equivalent current dipole analysis is that the investigator needs to decide rather subjectively on the number of dipoles fitted to the model. Choosing the wrong number of sources will increase the probability of an incorrect solution [44]. As a consequence, by seeding sources based on prior knowledge the source analysis improves objectivity [45]. Hopfinger, Khoe, and Song [23] have proposed a general methodological framework for combining fMRI and EEG data, and included four major aspects. They claimed that (1) the data should be based on an identical experimental frame including the timing, the instructions, response requirements and expectations of participants, which the present study covered. The present study also covered (2) the identical sensory frame including the same stimuli, and (3) the spatial reference using Talairach coordinates in both experiments. The only framework requirement, which was not covered by the present study was (4) the biological reference, i.e. identical sample of participants. However, gender and age were the same in both studies.

Second, several authors have stressed that source models can even be improved by additionally fitted sources because of possible mismatches between fMRI and EEG with regards to underlying activation patterns (see, e.g., [24], [26]).

Regional sources were seeded into a multiple discrete source model. RSs consist of three equivalent current dipoles at the same location with mutually orthogonal orientations [46], [47]. Thus, RSs represent neuronal current flow of arbitrary directions within the close range of the modeled brain region. As RS-moments are hardly susceptible to small differences between the modeled location of active brain regions and individual anatomical locations [46], [48], rather robust source waveforms should be obtained for the fMRI seeding technique despite anatomical differences between participants of the previous fMRI-study [27] and participants of the present EEG-study.

Regional source activity was analyzed separately for dynamic and static facial stimuli to study the respective modality-related neural dynamics of emotional category-related regional brain activations [47]. The procedure applied in the present study is analogous to the approach recently described by Bledowski and colleagues [45] and Wibral and colleagues [49].

Source waveforms were computed using a standard four-shell spherical head model, which takes into account conductance characteristics of brain, bone, cerebrospinal fluid, and scalp [46].

As there were different BOLD-activation patterns revealed for the processing of static and dynamic emotional faces [27], and because of the different inherently temporal characteristics of static and dynamic stimuli as derived from ERP data, two separate multiple source models were applied. Based on the respective fMRI-activation-patterns of the contrasts disgust>neutral and happiness>neutral [27] source models including seven generator-locations (four activation-foci for disgust and three activation-foci for happiness) for the static and 33 generator-locations (17 activation-foci for disgust and 16 activation-foci for happiness) for the dynamic modality were implemented. In order to reduce crosstalk of nearby sources (i.e., part of the variance in a source waveform is explained by activity generated at the location of all other sources [50] between adjacent regional cluster activations), Talairach coordinates of fMRI activation-foci being less than 30 mm apart from each other were combined according to the nearest neighbor method (for details, see [45]). Thus, closest pairs of Talairach coordinates were combined as long as the new coordinate did not exceed 20 mm to its original fMRI activation-focus. As described in previous studies, this approach appears to be appropriate because RS-waveforms are rather insensitive towards errors in equivalent center location of up to 20 mm [25], [45], [49]. This aspect was controlled in the current study because all RSs should have a minimal distance of 30 mm to each other. Only locations with an eccentricity of larger than 0.55 Polar/US were included in source models because brain regions deeper than this value produce rather small signals in EEG or sum up in a way that source moments result in invalidly high values as compared to rather superficial RS. Hence, limbic and posterior cingulate regions were excluded from the source model (see Tab. 1–2, lower part) - even though they substantially contributed to the emotion-specific differences as revealed by the corresponding previous fMRI-study [27].

**Table 1:** The extraction of RS locations by applying the nearest neighbor method for static stimuli.

static stimuli				
contrast activity	(x, y, z)	RS	brain region	(x, y, z)
L superior frontal gyrus	(−2, 5, 51)	RS1	L superior frontal gyrus	(−1, 4, 58)
L superior frontal gyrus	(−2, 5, 53)			
L superior frontal gyrus	(0, 3, 70)			
L precentral gyrus	(−40, −5, 61)	RS2	L precentral gyrus	(−40, −5, 61)
R cerebellar tonsil	(26, −56, −38)	RS3	L inferior frontal gyrus	(−46, 24, 17)
L putamen	(−20, 4, 0)	RS4	R cerebellar tonsil	(26, −56, −38)
		**additional RS**		
		RS5	L middle occipital gyrus	(−30, −91,2)
		RS6	R insula	(40, 5, 12)
		RS7	R fusiform gyrus	(39, −63, −9)
		RS8	L inferior temporal gyrus	(−49, −51, −11)
***excluded brain regions***		***eccentricity values***		
*L putamen*	*(*−*20, 4, 0)*	*ecc = .32*	*L putamen*	*(*−*20, 4, 0)*

Talairach coordinates (x,y,z [in millimeters]) of significant fMRI peak-activations and of the resulting pooled regional sources (RS) for static stimuli are presented. The lower part *(italic)* displays excluded brain areas due to eccentricity (ecc) values of ecc<.55. RS = regional sources, L = left; R = right.

**Table 2:** The extraction of RS locations by applying the nearest neighbor method for the dynamic stimulus modality.

dynamic stimuli					
contrast activity	(x, y, z)	RS	brain region		(x, y, z)
R lingual gyrus	(16, −90, −4)	RS1	R middle occipital gyrus, cuneus		(13, −90, 12)
R cuneus	(14, −95, 19)				
R cuneus	(10, −84, 30)				
L middle occipital gyrus	(−26, −97, 10)	RS2	L cuneus		(−25, −88, 25)
L cuneus	(−18, −92, 27)				
L precuneus	(−32, −74, 37)				
L supramarginal gyrus	(−53, −41, 35)	RS3	L superior temporal gyrus		(−49, −56, 29)
L superior temporal gyrus	(−50, −53, 21)				
L angular gyrus	(−44, −74, 31)				
R middle temporal gyrus	(55, −66, 3)	RS4	R superior temporal sulcus		(55, −52, 8)
R superior temporal gyrus	(67, −44, 13)				
R superior temporal gyrus	(46, −35, 5)				
R middle temporal gyrus	(51, −62, 12)				
R fusiform gyrus	(46, −67, −17)	RS5	R fusiform gyrus		(46, −67, −17)
L fusiform gyrus	(−42, −43, −15)	RS6	L fusiform gyrus		(−42, −59, −15)
L fusiform gyrus	(−42, −78, −15)				
L fusiform gyrus	(−42, −57, −16)				
R superior frontal gyrus	(6, 15, 62)	RS7	R superior frontal gyrus		(5, 18, 63)
R superior frontal gyrus	(4, 21, 63)				
L inferior frontal gyrus	(−55, 18, 6)	RS8	L precentral gyrus		(−42, 18, 7)
L extra-nuclear/claustrum	(−28, 18, 8)				
L medial frontal gyrus	(−10, 44, 25)	RS9	L medial frontal gyrus		(−7, 48, 11)
L medial frontal gyrus	(−4, 52, −4)				
R middle frontal gyrus	(59, 12, 36)	RS10	R inferior frontal gyrus		(61, 11, 26)
R inferior frontal gyrus	(63, 9, 16)				
R tuber (posterior vermis)	(24, −83, −29)	RS11	R tuber (posterior vermis)		(24, −83, −29)
		**additional RS**			
		RS12	R medial frontal gyrus		(11, −27, 57)
***excluded brain regions***		***eccentricity values***			
*R uncus*	*(18, 1,* −*20)*	*ecc = .26*	*R parahippocampal gyrus*		*(14,* −*8,* −*15)*
*R parahippocampal gyrus*	*(20,* −*12,* −*13)*				
*R mammillary body*	*(4,* −*12,* −*11)*				
*R posterior cingulate*	*(0,* −*54, 12)*	*ecc = .36*	*R posterior cingulate cortex*		*(9,* −*49, 8)*
*R parahippocampal gyrus*	*(18,* −*43, 4)*				
*L uncus*	*(*−*24, 4,* −*34)*	*ecc = .53*	*L uncus*		*(*−*24, 4,* −*34)*
*R hippocampus*	*(*−*28,* −*29,* −*7)*	*ecc = .38*	*R hippocampus*		*(*−*28,* −*29,* −*7)*

Talairach coordinates (x,y,z [in millimeters]) of significant fMRI activation foci and of the resulting pooled regional sources (RS) for dynamic stimuli are presented. One additional RS (RS 12) was seeded for the dynamic source model. The lower part *(italic)* displays excluded brain areas due to eccentricity (ecc) values of ecc<.55. RS = regional sources, L = left; R = right.

**Figure 3:**
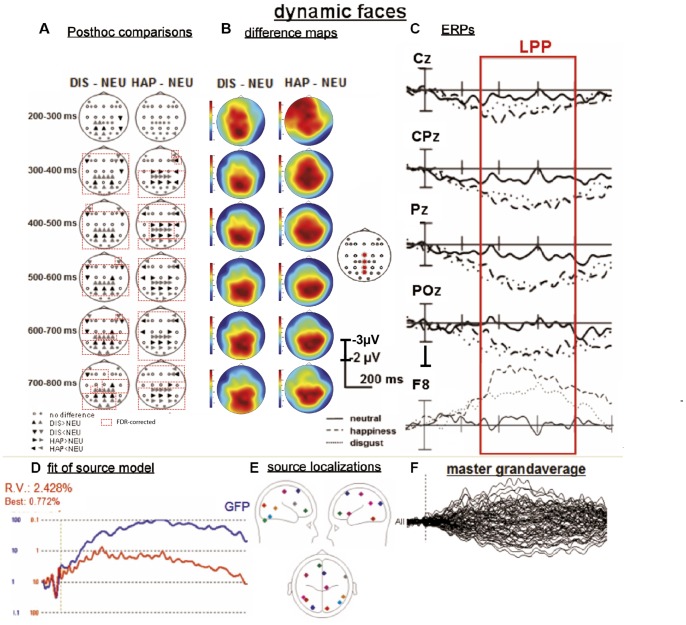
Results of ERP and fMRI constrained source analysis of dynamic facial expressions. (A) posthoc comparisons for six selected time windows (dependent t-tests, p<.05). Dashed red boxes represent significant results after FDR-correction. (B) Topographical maps of difference waves for disgust (DIS)>neutral (NEU) and happiness (HAP)>NEU and neutral alone. (C) ERPs for selected posterior midline and anterio-lateral electrode positions. Red box indicates the analyzed time window of the LPP. Source model of dynamic stimuli: (F) master grand-average over all electrodes and all conditions (−100–1000 ms), (D) Global field power curve (GFP; blue curve) and explained variance of the model (residual variance [RV] and best fit; red curve), (E) Twelve RS (each displayed in individual color) projected onto a 4-shell spherical model displayed from six different perspectives (saggital [left, right], transversal [above, below], coronal [back, front]). Note: RS one to eleven were seeded based on fMRI activations and RS twelve was added by sequential fitting procedure.

Finally, four RSs for the static and eleven RSs for the dynamic modality were seeded into two different source models, which were applied on ERP-data (post-stimulus interval: 0–1000 ms, 30 Hz low pass filtered). The resulting source models were firstly applied on the master grand average over all emotion-related stimulus conditions (neutral, happiness, and disgust, separately for static and dynamic modality, Fig. 2D and 3D, respectively), A sequential fitting procedure (as described in BESA® Tutorial, Chapter 3: Tutorial of EEG reaction time experiment) was applied in order to saturate each modality-related source model while reducing the residual variance step-wise below five percent. Thus, the final source models should explain at least 95% of the variance.

Time epochs of interest for fitting additional sources for the static modality were identified after visual inspection of the time courses of the residual variance (Fig. 2E, red curve), the global field power curve (see Fig. 2E, blue curve), and the master grand average (Fig. 2D). The following RSs were fitted in the following time epochs: (1) two RSs were fitted in an early epoch from 52 to 113 ms, (2) two RSs were fitted in a later epoch from 191 to 462 m, (3) and one RS was fitted over a long epoch from 68 to 996 ms. Finally, the seeded model for the static modality resulted in a regional source model, which included eight discrete RSs (see Fig. 2F, Tab. 1) explaining a common variance of 99.3% (see Fig. 2 E).

For the dynamic modality, the seeded source model included eleven RSs explaining 96.7% of common variance. Only one additional source was added to the model for a time interval from 95 to 951 ms. The attempt to fit additional sources resulted in source locations lying outside of the head, which was considered as exclusion criteria. Thus, the final seeded model for the dynamic modality resulted in a model including eleven seeded RSs and one additionally fitted discrete RS (Fig. 3F, Tab. 2) explaining a common variance of 97.6% (Fig. 3E).

The root mean square (RMS) curve of each RS (calculated as the square root of the mean of the added and squared source moment [in nAm] of three mutually orthogonal dipoles per generator location) for each emotional stimulus condition and each participant was calculated using BESA®-Software and exported for further analyses.

**Figure 4:**
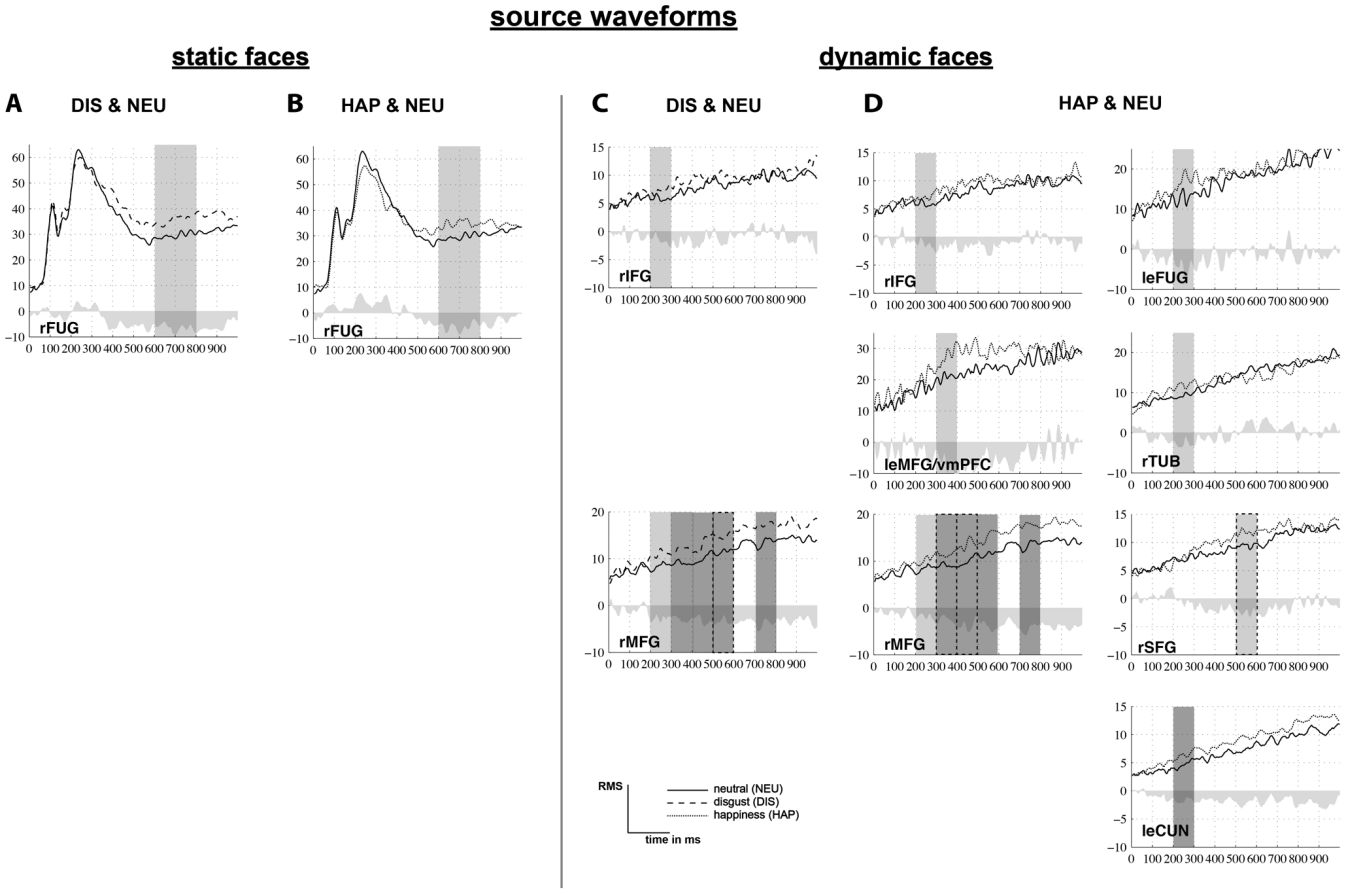
Source waveforms of significantly different RS-activations. Source waveforms (root mean square (RMS) curve of each regional source) are displayed over time (0-1000ms) for each condition (solid line  =  neutrality (NEU), dashed line  =  disgust (DIS), dotted line =  happiness (HAP). Significant RS-activations are based on ANOVAs (light grey: significant at p<.05, dark grey: trend to significance at p<.1) and post hoc comparisons (box: no frame: p<.05, dashed frame: p<.1) for static (A) DIS > NEU, (B) HAP > NEU and dynamic facial expressions (C) DIS > NEU (D) HAP > NEU. Abbreviations: le = left, r = right, CUN  =  cuneus, FUG  =  fusiform gyrus, IFG  =  inferior frontal gyrus, MFG  =  medial frontal gyrus, SFG  =  superior frontal gyrus, TUB  =  tuber, vmPFC  =  ventromedial prefrontal cortex (medial frontal gyrus).

Based on our apriori knowledge of the fMRI constraints and knowledge of the ERP time windows of interest, we decided to calculate a region of interest (ROI) analysis. To investigate potential time course differences of the emotional stimulus category-related source waveforms, mean-amplitudes of the respective RMS-source-moment-values (plotted in Fig. 4) were calculated for the apriori defined ERP time windows for each RS and each individual via inferential statistics (referred to as “source activity” in the following paragraphs).

Our ROI analysis consisted of repeated measurement ANOVAs, which were performed on mean amplitude source activity values (separately for static and dynamic modality) for three different time windows (N170, EPN, LPP) for static, and six 100 ms time windows (from 200–800 ms) for dynamic stimulus modality separately. ANOVAs included the within-subject factors CATEGORY (CAT: three levels: Neutral, happiness, disgust). Post hoc analyses (paired sample t-tests, uncorrected) were calculated according to significant or trend to significant main effects. Posthoc comparisons were not corrected for multiple comparisons because ROI analysis was based on a priori knoweledge in both the temporal and spatial domain.

## Results

### Behavioral Data

Post-hoc evaluation of emotional stimuli revealed a categorization accuracy for static faces of 93.7% (SD: 4.6%) for neutral, 96.7% (SD: 4.3%) for happiness, and 95.7% (SD: 3.1%) for disgust, and for the dynamic faces of 92.1% (SD: 12.7%) for neutral, 98.0% (SD: 3.5%) for happiness, and 96.2% (SD: 3.9%) for disgusted expressions. Categorization accuracy was not statistically different between static and dynamic stimuli in post-hoc comparisons.

Arousal showed a main effect of CATEGORY (CAT; F_[2,34]_ = 22.2, p<.001; explained by higher arousal for emotional compared to neutral facial expressions) and of MODALITY (MOD; F_[1,17]_ = 7.2, p<.05; higher arousal for static compared to dynamic stimuli). The CAT×MOD interaction (F_[2,34]_ = 3.8, p = .03) was explained by significantly higher arousal rates for static compared to dynamic emotional stimuli (paired sample t-tests: p<.05 for happiness, p<.01 for disgust).

### ERP Data - Static Faces

In the static stimulus modality, for three time windows, three-way interactions (ANTERIOR-POSTERIOR (AP)×LATERALITY (LAT)×CATEGORY (CAT)) reached significance or a trend to significance (N170: F_[6.6,116.4]_ = 2.0, p = .063, EPN: F_[6.2,111.6]_ = 3.5, p<.01, LPP: F_[7.8, 140.6]_ = 2.0, p<.01, all Greenhouse-Geisser (GG-)adjusted). Additional ANOVAs for the three different time windows with the factor CATEGORY (CAT, three levels: neutral, happiness, disgust) and posthoc comparisons were calculated for several electrode-positions of interest (e.g., PO7/8 or O1/2 etc., see grey filled circles displayed in Fig. 2A) in order to statistically prove potential effects of the topographical analysis in surrounding electrode sites.

Post-hoc t-tests revealed that the N170 component yielded a larger mean amplitude value for disgust compared to neutral stimuli at electrode position P8, and for happiness compared to neutral facial expressions at electrode position PO8 (Fig. 2A and C, Tab. 3). This effect is illustrated by topographic voltage maps showing an increased right lateralized posterior negative scalp distribution for happiness and a bilateral posterior negative scalp distribution for disgust (Fig. 2A). Post-hoc tests related to the EPN-epoch revealed a relative negativity for disgust compared to neutral faces at posterior temporo-occipital electrode sites (P8, PO8, O2; see Fig. 2A), as illustrated by the respective scalp voltage maps (Fig. 2B). In addition, electrode F8, as well as F7 and T7, sh owed an enhanced positivity for disgusted compared to neutral static expressions in the same time window (see Fig. 2C, dashed line in blue box for respective ERP time window, 2B for topographical voltage maps, and 2A for an illustration of the post-hoc comparisons). Enhanced positive mean amplitudes of the LPP for disgust (bilateral at P3, Pz, and P4) and happy (lateralized to the left at P3 and Pz) compared to neutral stimuli between (600–800 ms) at parietal electrodes sites were illustrated by voltage maps showing an enhanced positivity over midline parietal regions (Fig. 2B and C). For FDR-corrected results please refer to Fig. 2A (dashed red boxes, and Tab. 3, p-values highligted in bold). Only LPP for disgust compared to neutral facial expressions remained significant after FDR-correction on electrodes Pz, P3, and P4.

**Table 3:** Post-hoc comparisons of ERPs for static facial expressions for N170, EPN and LPP.

static stimuli						
*ms*	*ERP*	*comparison*	*electrode*	*T*	*df*	*p*
140–190	N170	neu-dis	P8	2.11	18	.050
		*neu-dis*	*PO8*	*2,42*	*18*	.*026*
250–350	EPN	neu-dis	P8	2,35	18	,031
		neu-dis	O2	2,30	18	,034
		neu-dis	F7	−3,24	18	,005
		neu-dis	F8	−2,42	18	,026
		hap-dis	F8	−2,36	18	,030
		hap-dis	F7	−2,98	18	,008
		hap-dis	T7	−2,46	18	,024
		*neu-dis*	*PO8*	*2,61*	*18*	,*018*
600–800	LPP	neu-dis	P4	−3,18	18	**,005**
		neu-dis	Pz	−3,45	18	**,003**
		neu-dis	P3	−3,23	18	**,005**
		neu-hap	Pz	−2,70	18	,015
		neu-hap	P3	−2,27	18	,036

Table displays significant post-hoc comparisons (paired t-tests) for static facial expressions including the time window (ms), ERP-component (N170, EPN, LPP), the kind of comparison (neu = neutral, hap = happy, dis = disgusted), electrode site, T-values, degrees of freedom (df), and p-values (p-values highligted in bold remain significant after FDR correction). Grey and italic font represents additional electrodes of interests (see methods for further explanations).

### ERP Data - Dynamic Faces

Main effects or interactions including the factor CATEGORY were identified for all seven time windows examined for the dynamic facial expression modality (GG-adjustment, where appropriate, Tab. 4, Fig. 3). Additional ANOVAs for the seven different time windows with the factor CATEGORY (CAT, three levels: neutral, happiness, disgust) were calculated for several electrode-positions of interest (e.g., CPz, FCz etc., see grey filled circles displayed in Fig. 3A) in order to statistically substantiate potential effects of the topographical analysis in surrounding electrode sites.

**Table 4:** Topographical analyses of emotional category effects in the dynamic emotional stimulus modality.

Dynamic stimuli					
Ms	Factors	*df*	F	p	GG
100–200	AP	2, 36	7.68	<.01	<.01*
100–200	LAT	4, 72	8.77	<.01	<.01*
100–200	APxLAT	8, 144	6.63	<.01	<.01
100–200	APxLATxCAT	16, 288	1.82	<.05	<.1*
200–300	AP	2, 36	4.24	<.05	<.05*
200–300	LAT	4, 72	17.66	<.01	<.01*
200–300	APxLAT	8, 144	6.17	<.01	<.01*
200–300	LATxCAT	8, 144	3.10	<.01	<.05*
300–400	AP	2, 36	7.84	<.01	<.01*
300–400	LAT	4, 72	22.14	<.01	<.01*
300–400	APxLAT	8, 144	3.35	<.01	<.05*
300–400	LATxCAT	8, 144	6.15	<.01	<.01*
400–500	AP	2, 36	18.26	<.01	<.01*
400–500	LAT	4, 72	17.62	<.01	<.01*
400–500	CAT	2, 36	2.80	<.1	<.1
400–500	APxLAT	8, 144	3.99	<.01	<.01*
400–500	APxCAT	4, 72	3.44	<.05	<.05*
400–500	LATxCAT	8, 144	6.09	<.01	<.01*
500–600	AP	2, 36	18.87	<.01	<.01*
500–600	LAT	4, 72	11.55	<.01	<.01*
500–600	CAT	2, 36	1.52	<.01	<.01
500–600	APxLAT	8, 144	3.88	<.01	<.01*
500–600	APxCAT	4, 72	7.73	<.01	<.01*
500–600	LATxCAT	8, 144	5.64	<.01	<.01*
600–700	AP	2, 36	10.65	<.01	<.01*
600–700	LAT	4, 72	7.00	<.01	<.01*
600–700	APxLAT	8, 144	3.28	<.01	<.01*
600–700	APxCAT	4, 72	6.25	<.01	<.01*
600–700	LATxCAT	8, 144	5.42	<.01	<.01*
700–800	AP	2, 36	6.94	<.01	<.05*
700–800	LAT	4, 72	4.94	<.01	<.05*
700–800	APxLAT	8, 144	2.95	<.01	<.05*
700–800	APxCAT	4, 72	4.31	<.01	<.05*
700–800	LATxCAT	8, 144	3.67	<.01	<.05*

Statistical parameters (df = degrees of freedom, F = F-value, p = p-value, GG = GG-adjusted p-value, * = GG-correction appropriate) of repeated measurement ANOVAs including mean amplitude values of 15 equidistant electrode positions pooled for the two topographical factors ANTERIOR-POSTERIOR (AP: Three levels) and LATERALITY (LAT: Five levels), and the factor EMOTIONAL CATEGORY (CAT: Three levels) separately for seven different time windows of the respective ERPs.

Post-hoc t-tests were calculated whenever the factor CATEGORY was included in a significant or trend to significant interaction (Tab. 4). Post-hoc tests showed enhanced positivity in both disgust (after 200 ms) and happy (after 300 ms) compared to neutral stimuli at central, centro-parietal, parietal, and parieto-occipital electrodes (see Fig. 3A and Tab. 5, for post-hoc comparisons and Fig. 3B for the respective scalp topographies). For FDR-corrected results please refer to Fig. 3A (dashed red boxes, and Table 5, p-values highligted in bold). Except for results of the the time window 200–300 ms and a few electrode sites for the following time windows (see Tab. 5, non-bold p-values, and Fig. 3 A, electrode sites not included in red dashed boxes), most of the reported non-corrected results survived FDR-correction and remained significant (see Tab. 5, p-values highligted in bold).

**Table 5:** Post-hoc comparisons of ERPs for dynamic facial expressions for component LPP.

dynamic stimuli						
*ms*	*ERP*	*comparison*	*electrode*	*T*	*df*	*p*
200–300	LPP	neu-hap	P8	3,404	18	,003
		neu-dis	F8	2,397	18	,028
		neu-dis	T8	3,078	18	,006
		neu-dis	P8	2,196	18	,041
		neu-dis	Pz	−3,147	18	,006
		neu-dis	P3	−2,347	18	,031
		*neu-hap*	*PO8*	*2,482*	*18*	,*023*
		*neu-dis*	*CP3*	−*2,359*	*18*	,*030*
		*neu-dis*	*CP1*	−*2,275*	*18*	,*035*
		*neu-dis*	*PO3*	−*2,582*	*18*	,*019*
		*neu-dis*	*POz*	−*2,806*	*18*	,*012*
		*hap-dis*	*PO3*	−*2,428*	*18*	,*026*
		*hap-dis*	*O2*	−*2,391*	*18*	,*028*
		*hap-dis*	*O1*	−*2,105*	*18*	,*050*
300–400	LPP	neu-hap	F8	3,658	18	**,002**
		neu-hap	T8	3,313	18	**,004**
		neu-hap	Cz	−3,021	18	**,007**
		neu-hap	C3	−2,328	18	**,032**
		neu-hap	P8	2,809	18	**,012**
		neu-hap	P4	−2,843	18	**,011**
		neu-hap	Pz	−3,156	18	**,005**
		neu-hap	P3	−2,353	18	**,030**
		neu-dis	P3	−3,383	18	**,003**
		neu-dis	F8	3,469	18	**,003**
		neu-dis	F7	2,542	18	**,020**
		neu-dis	T8	3,049	18	**,007**
		neu-dis	P4	−3,194	18	**,005**
		neu-dis	Pz	−5,448	18	**,000**
		*neu-hap*	*CP3*	−*3,022*	*18*	**,** ***007***
		*neu-hap*	*CP1*	−*2,980*	*18*	**,** ***008***
		*neu-hap*	*CPz*	−*3,195*	*18*	**,** ***005***
		*neu-hap*	*CP2*	−*3,147*	*18*	**,** ***006***
		*neu-hap*	*CP4*	−*2,959*	*18*	**,** ***008***
		*neu-hap*	*AF8*	*2,972*	*18*	**,** ***008***
		*neu-hap*	*F6*	*2,302*	*18*	**,** ***033***
		*neu-hap*	*F6*	*2,237*	*18*	,*038*
		*neu-dis*	*CP3*	−*3,224*	*18*	**,** ***005***
		*neu-dis*	*CP1*	−*3,034*	*18*	**,** ***007***
		*neu-dis*	*CPz*	−*2,796*	*18*	**,** ***012***
		*neu-dis*	*CP2*	−*2,918*	*18*	**,** ***009***
		*neu-dis*	*CP4*	−*2,334*	*18*	**,** ***031***
		*neu-dis*	*PO3*	−*4,680*	*18*	**,** ***000***
		*neu-dis*	*PO4*	−*4,585*	*18*	**,** ***000***
		*neu-dis*	*PO4*	−*3,792*	*18*	**,** ***001***
		*neu-dis*	*AF8*	*2,818*	*18*	**,** ***011***
		*hap-dis*	*PO8*	−*2,360*	*18*	**,** ***030***
		*hap-dis*	*O2*	−*2,477*	*18*	**,** ***023***
400–500	LPP	neu-hap	F8	4,559	18	**,000**
		neu-hap	F7	2,422	18	**,026**
		neu-hap	T8	2,242	18	,038
		neu-hap	C4	−2,552	18	**,020**
		neu-hap	C4	−2,778	18	**,012**
		neu-hap	C3	−3,363	18	**,003**
		neu-hap	P4	−3,961	18	**,001**
		neu-hap	Pz	−4,345	18	**,000**
		neu-hap	P3	−3,615	18	**,002**
		neu-dis	F8	2,674	18	**,015**
		neu-dis	F7	3,205	18	**,005**
		neu-dis	P4	−4,481	18	**,000**
		neu-dis	Pz	−5,227	18	**,000**
		neu-dis	P3	−3,565	18	**,002**
		hap-dis	F8	−2,465	18	**,024**
		*neu-hap*	*CP3*	−*4,535*	*18*	**,** ***000***
		*neu-hap*	*CP1*	−*4,603*	*18*	**,** ***000***
		*neu-hap*	*CPz*	−*4,364*	*18*	**,** ***000***
		*neu-hap*	*CP2*	−*3,669*	*18*	**,** ***002***
		*neu-hap*	*CP4*	−*3,683*	*18*	**,** ***002***
		*neu-hap*	*PO3*	−*2,400*	*18*	**,** ***027***
		*neu-hap*	*POz*	−*2,577*	*18*	**,** ***019***
		*neu-hap*	*PO4*	−*2,623*	*18*	**,** ***017***
		*neu-hap*	*AF7*	*2,856*	*18*	**,** ***010***
		*neu-hap*	*AF8*	*4,273*	*18*	**,** ***000***
		*neu-hap*	*F6*	*2,971*	*18*	**,** ***008***
		*neu-dis*	*CP3*	−*3,519*	*18*	**,** ***002***
		*neu-dis*	*CP1*	−*2,980*	*18*	**,** ***008***
		*neu-dis*	*CPz*	−*2,891*	*18*	**,** ***010***
		*neu-dis*	*CP2*	−*2,935*	*18*	**,** ***009***
		*neu-dis*	*CP4*	−*2,338*	*18*	**,** ***031***
		*neu-dis*	*PO3*	−*3,817*	*18*	**,** ***001***
		*neu-dis*	*POz*	−*3,607*	*18*	**,** ***002***
		*neu-dis*	*PO4*	−*4,116*	*18*	**,** ***001***
		*neu-dis*	*AF8*	*2,224*	*18*	,*039*
		*hap-dis*	*CP3*	*2,224*	*18*	,*039*
500–600	LPP	neu-hap	F8	4,472	18	**,000**
		neu-hap	F7	3,559	18	**,002**
		neu-hap	C4	−4,481	18	**,000**
		neu-hap	C3	−2,460	18	,024
		neu-hap	T7	2,585	18	**,019**
		neu-hap	P4	−5,088	18	**,000**
		neu-hap	Pz	−4,475	18	**,000**
		neu-hap	P3	−3,450	18	**,003**
		neu-dis	F8	2,719	18	**,014**
		neu-dis	F7	3,745	18	**,001**
		neu-dis	C4	−2,665	18	**,016**
		neu-dis	P4	−5,793	18	**,000**
		neu-dis	Pz	−5,639	18	**,000**
		neu-dis	P3	−3,836	18	**,001**
		*neu-hap*	*AF7*	*3,404*	*18*	**,** ***003***
		*neu-hap*	*AF8*	*3,793*	*18*	**,** ***001***
		*neu-hap*	*CP1*	−*3,577*	*18*	**,** ***002***
		*neu-hap*	*CP2*	−*4,610*	*18*	**,** ***000***
		*neu-hap*	*CP3*	−*4,206*	*18*	**,** ***001***
		*neu-hap*	*CP4*	−*5,951*	*18*	**,** ***000***
		*neu-hap*	*CPz*	−*4,125*	*18*	**,** ***001***
		*neu-hap*	*F6*	*3,654*	*18*	**,** ***002***
		*neu-hap*	*PO3*	−*3,518*	*18*	**,** ***002***
		*neu-hap*	*PO4*	−*3,673*	*18*	**,** ***002***
		*neu-hap*	*POz*	−*3,095*	*18*	**,** ***006***
		*neu-dis*	*AF7*	*2,142*	*18*	,*046*
		*neu-dis*	*AF8*	*2,403*	*18*	**,** ***027***
		*neu-dis*	*CP1*	−*2,783*	*18*	**,** ***012***
		*neu-dis*	*CP2*	−*3,715*	*18*	**,** ***002***
		*neu-dis*	*CP3*	−*3,108*	*18*	**,** ***006***
		*neu-dis*	*CP4*	−*3,364*	*18*	**,** ***003***
		*neu-dis*	*CPz*	−*3,440*	*18*	**,** ***003***
		*neu-dis*	*F6*	*2,565*	*18*	**,** ***019***
		*neu-dis*	*O1*	−*2,539*	*18*	**,** ***021***
		*neu-dis*	*O2*	−*3,268*	*18*	**,** ***004***
		*neu-dis*	*PO3*	−*3,780*	*18*	**,** ***001***
		*neu-dis*	*PO4*	−*4,896*	*18*	**,** ***000***
		*neu-dis*	*PO8*	−*2,109*	*18*	,*049*
		*neu-dis*	*POz*	−*3,772*	*18*	**,** ***001***
		*hap-dis*	*CP4*	*2,161*	*18*	,*044*
600–700	LPP	neu-hap	F8	4,327	18	**,000**
		neu-hap	F7	2,741	18	**,013**
		neu-hap	C4	−3,677	18	**,002**
		neu-hap	T7	2,527	18	**,021**
		neu-hap	P4	−4,073	18	**,001**
		neu-hap	Pz	−4,022	18	**,001**
		neu-hap	P3	−2,986	18	**,008**
		neu-dis	F8	3,106	18	**,006**
		neu-dis	F7	3,603	18	**,002**
		neu-dis	T8	2,179	18	,043
		neu-dis	C4	−2,542	18	**,020**
		neu-dis	T7	2,316	18	**,033**
		neu-dis	P4	−3,929	18	**,001**
		neu-dis	Pz	−4,855	18	**,000**
		neu-dis	P3	−3,381	18	**,003**
		neu-dis	P7	2,354	18	**,030**
		*neu-hap*	*AF7*	*2,433*	*18*	**,** ***026***
		*neu-hap*	*AF8*	*3,683*	*18*	**,** ***002***
		*neu-hap*	*CP1*	−*3,243*	*18*	**,** ***005***
		*neu-hap*	*CP2*	−*4,230*	*18*	**,** ***001***
		*neu-hap*	*CP3*	−*3,356*	*18*	**,** ***004***
		*neu-hap*	*CP4*	−*4,704*	*18*	**,** ***000***
		*neu-hap*	*CPz*	−*3,711*	*18*	**,** ***002***
		*neu-hap*	*F6*	*3,503*	*18*	**,** ***003***
		*neu-hap*	*PO3*	−*3,400*	*18*	**,** ***003***
		*neu-hap*	*PO4*	−*3,169*	*18*	**,** ***005***
		*neu-hap*	*POz*	−*3,301*	*18*	**,** ***004***
		*neu-dis*	*AF7*	*2,135*	*18*	,*047*
		*neu-dis*	*AF8*	*2,329*	*18*	**,** ***032***
		*neu-dis*	*CP1*	−*2,889*	*18*	**,** ***010***
		*neu-dis*	*CP2*	−*3,724*	*18*	**,** ***002***
		*neu-dis*	*CP3*	−*2,895*	*18*	**,** ***010***
		*neu-dis*	*CP4*	−*2,860*	*18*	**,** ***010***
		*neu-dis*	*CPz*	−*3,910*	*18*	**,** ***001***
		*neu-dis*	*F6*	*2,472*	*18*	**,** ***024***
		*neu-dis*	*O1*	−*3,225*	*18*	**,** ***005***
		*neu-dis*	*O2*	−*3,141*	*18*	**,** ***006***
		*neu-dis*	*PO3*	−*4,249*	*18*	**,** ***000***
		*neu-dis*	*PO4*	−*4,489*	*18*	**,** ***000***
		*neu-dis*	*POz*	−*3,755*	*18*	**,** ***001***
		*hap-dis*	*CP4*	*2,152*	*18*	,*045*
700–800	LPP	neu-hap	F8	3,506	18	**,003**
		neu-hap	C4	−3,178	18	**,005**
		neu-hap	C3	−2,108	18	,049
		neu-hap	P4	−3,500	18	**,003**
		neu-hap	Pz	−3,469	18	**,003**
		neu-hap	P3	−2,634	18	**,017**
		neu-dis	PO8	−2,137	18	,047
		neu-dis	F7	3,464	18	**,003**
		neu-dis	C4	−2,545	18	**,020**
		neu-dis	P4	−4,467	18	**,000**
		neu-dis	Pz	−3,990	18	**,001**
		neu-dis	P3	−2,457	18	**,024**
		neu-dis	O2	−3,594	18	**,002**
		neu-dis	O1	−2,378	18	,029
		hap-dis	PO8	−2,906	18	**,009**
		hap-dis	F7	2,512	18	**,022**
		hap-dis	O2	−3,173	18	**,005**
		*neu-hap*	*AF8*	*2,433*	*18*	**,** ***026***
		*neu-hap*	*CP1*	−*2,860*	*18*	**,** ***010***
		*neu-hap*	*CP2*	−*3,475*	*18*	**,** ***003***
		*neu-hap*	*CP3*	−*4,065*	*18*	**,** ***001***
		*neu-hap*	*CP4*	−*3,687*	*18*	**,** ***002***
		*neu-hap*	*CPz*	−*3,040*	*18*	**,** ***007***
		*neu-hap*	*F6*	*2,802*	*18*	**,** ***012***
		*neu-hap*	*PO3*	−*2,901*	*18*	**,** ***010***
		*neu-hap*	*PO4*	−*2,308*	*18*	**,** ***033***
		*neu-hap*	*POz*	−*2,676*	*18*	**,** ***015***
		*neu-dis*	*CP1*	−*2,214*	*18*	,*040*
		*neu-dis*	*CP2*	−*3,331*	*18*	**,** ***004***
		*neu-dis*	*CP3*	−*2,617*	*18*	**,** ***017***
		*neu-dis*	*CP4*	−*2,773*	*18*	**,** ***013***
		*neu-dis*	*CPz*	−*2,994*	*18*	**,** ***008***
		*neu-dis*	*O6*	−*2,378*	*18*	**,** ***029***
		*neu-dis*	*O2*	−*3,594*	*18*	**,** ***002***
		*neu-dis*	*PO3*	−*2,855*	*18*	**,** ***011***
		*neu-dis*	*PO4*	−*4,863*	*18*	**,** ***000***
		*neu-dis*	*PO8*	−*2,137*	*18*	,*047*
		*neu-dis*	*POz*	−*3,372*	*18*	**,** ***003***
		*hap-dis*	*O2*	−*3,173*	*18*	**,** ***005***
		*hap-dis*	*PO8*	−*2,906*	*18*	**,** ***009***

Table displays significant post-hoc comparisons (paired t-tests) for dynamic facial expressions including the different time windows (ms), ERP-component (LPP), the kind of comparison (neu = neutral, hap = happy, dis = disgusted), electrode site, T-values, degree of freedom (df), and p-values (p-values highligted in bold remain significant after FDR correction). Grey and italic font represents additional electrodes of interests (see methods for further explanations).

### fMRI Constrained Source Analysis - Static Facial Stimuli

As revealed by prior fMRI examinations (Trautmann et al., 2009), the seeded source model for static facial expressions encompassed several source locations including the superior, inferior frontal, precentral gyrus, and the cerebellar tonsil. This model was extended by additional fitting procedures yielding sources located at in middle occipital gyrus, insula, and inferior temporal gyrus including the FuG (for illustration of source locations, see Fig. 2F).

Region of interest (ROI) analysis of significant main effects (see light grey boxes in Fig. 4 A and B) of repeated-measurement ANOVAs including emotional CATEGORY as factor are listed in Table 6 A for a priori defined ERP time windows of the N170 and LPP. Results of post hoc comparisons are illustrated in Fig. 4 (A and B, sourcewaveforms), and Tab. 6 A (column: posthoc).

**Table 6:** Regional source analyses of emotional category effects in the static and dynamic stimulus modality.

(A) static stimuli								
*TW*	*RS*	*label (TAL)*	*factor*	*df*	*F*	*p*	*GG*	*posthoc*
*N170*	*4*	*rCerTON*	*EMO*	*2,36*	*2.8*	*<.1*	*<.1*	
	*7*	*rFUG*	*EMO*	*2,36*	*2.7*	*<.1*	*<.1*	dis>hap
LPP	7	rFUG	EMO	2,36	3.5	<.05	<.05	hap>neu,dis>neu
**(B) dynamic stimuli**								
200–300 ms	6	leFUG	EMO	2, 36	4.5	<.05	<.05	hap>neu, hap>dis
	10	rIFG	EMO	2, 36	3.5	<.05	<.05	hap>neu, dis>neu
	11	rTUB	EMO	2, 36	3.4	<.05	<.05	hap>neu, hap>dis
	12	rMFG	EMO	2, 36	4.0	<.05	<.05	dis>neu, hap>neu
	*2*	*leCUN*	*EMO*	*2, 36*	*2.7*	.*08*	.*08*	*hap>neu*
300–400 ms	9	leMFG/vmPFC	EMO	2, 36	5.8	<.05		hap>neu, hap>dis
	*12*	*rMFG*	*EMO*	*2, 36*	*2.9*	.*07*	.*07*	dis>neu, *hap>neu*
400–500 ms	9	leMFG/vmPFC	EMO	2, 36	6.9	<.01	<.01	hap>neu, hap>dis
	*12*	*rMFG*	*EMO*	*2, 36*	*3.1*	.*06*	.*06*	dis>neu, *hap>neu*
500–600 ms	7	rSFG	EMO	2, 36	3.2	.05	.05	hap>dis, *hap>neu*
	11	rTUB	EMO	2, 36	4.2	<.02	<.02	dis<neu, *hap>dis*
	*12*	*rMFG*	*EMO*	*2, 36*	*2.6*	.*09*	.*09*	hap>neu,*dis>neu*
*700–800 ms*	*12*	*rMFG*	*EMO*	*2, 36*	*3.0*	*<.06*	*<.06*	hap>neu, dis>neu

Repeated measurement ANOVAs of region of interest (ROI) analysis of source analysis based on time windows (TW) of the ERP analysis for (A) static (N170, LPP) and (B) dynamic facial (five out of 6 100 ms time windows) expressions including the within-subject *factor* EMOTION (EMO, three levels: Neutral, happiness, disgust). Furthermore, regional sources (RS), RS labels (TAL), degrees of freedom (*df*), F-value (*F*), significance level (*p*), Greenhouse-Geisser adjusted p-values (*GG*), and posthoc (dependent t-tests, p<.05, or trend p<.1 in grey and italic) are depicted are depicted. Grey and italic text represents trend to statistical significance (p<.1). Asterics (*) indicate that GG-correction was applied. Abbreviations for anatomical RS-positions in order of appearance: r = right, le = left, CerTON = cerebellar tonsil, FUG = fusiform gyrus, IFG = inferior frontal gyrus, TUB = tuber, MFG = medial frontal gyrus, vmPFC = ventromedial prefrontal gyrus, CUN = cuneus, SFG = superior frontal gyrus; for Talairach coordinates and more details, see also Tab. 1 and 2.

ROI analysis of a priori defined ERP time windows resulted in enhanced source activity for disgust compared to neutral and for happy compared to neutral static stimulus processing between 600 and 800 ms (time window of the LPP) in right fusiform gyrus (FUG, see Tab. 6 A and Fig. 4 A and B). Hence, the underlying source of the emotion-specific ERP effects of the LPP is predominantly represented by the right FUG.

ROI analysis of the N170 showed a trend to significance in right FUG with an enhanced N170 for disgust compared to happiness) and in cerebellar tonsil (no posthoc comparisons reached statistical significance). ROI analysis of the EPN did not reach statistical significance.

In summary, only a posterior regional source reached significant different source activation between emotional and neutral facial expressions in a late time window for the static stimulus modality.

### fMRI Constrained Source Analysis – Dynamic Facial Stimuli

The source model arranged for the dynamic modality was based on twelve RSs, of which eleven were based on activation patterns revealed by the prior fMRI study (Trautmann et al., 2009), and one additional RS was fitted to the model (see Fig. 3 D–F for an illustration of the respective source analysis). RS-locations refer to occipital areas, superior temporal, inferior temporal (close to the fusiform face area and the fusiform gyrus), superior frontal, pre-central, ventral medial, and inferior frontal areas, and tuber (posterior vermis) of the cerebellum.

Region of interest (ROI) analysis on each regional source (RS) over the six predefined ERP time windows showed significant main effects (see light grey boxes in Fig. 4 C and D) or trends to significance (see dark grey boxes in Fig. 4 C and D) in repeated-measurement ANOVAs including emotional CATEGORY as factor (see Table 6 B). Results of post hoc comparisons are depicted in Tab. 6 B (column: posthoc) and are illustrated by means of sourcewaveforms in Fig. 4 (C and D).

The perceptual processing of disgust compared to neutral dynamic stimuli produced significantly larger source moment values (all p<.05, unless differently indicated) in right inferior frontal gyrus (IFG, 200–300 ms) and right medial frontal gyrus (MFG, 200–300 ms). Furthermore, continuous enhanced source activity was shown in right medial frontal gyrus between 300–500 ms and 700–800 ms, as well as between 500–600 ms (based on trends to significance in posthoc comparisons, see dashed boxes in Fig. 4 D, and should therefore be handled with care; Tab. 6 B, Fig. 4 C).

Happiness compared to neutral dynamic stimuli produced significantly enhanced source activity (all p<.05, unless differently indicated) in right inferior frontal gyrus (IFG), left cuneus. left fusiform gyrus (FUG), and right tuber (posterior vermis) between 200 and 300 ms (Tab. 6 B, Fig. 4 D). Left ventral medial frontal gyrus (MFG, ventromedial prefrontal cortex) showed enhanced source activity between 300 and 400 ms. Right MFG showed a trend to significance (see dashed boxes in Fig. 4 D) of enhanced source activity fom happy compared to neutral facial expressions within the same time window. Right MFG showed continuously higher source activity (ANOVAs showed trend to significance, while posthoc comparisons did not between 500–600 and 700–800 ms) up to 800 ms, as well as right superior frontal gyrus (SFG, 500–600 ms; Tab. 6 B, Fig. 4 D).

In summary, both posterior and anterior brain regions showed emotion-specific effects for the dynamic stimulus modality.

## Discussion

In the present study, dynamics of brain activation during the processing of visual static and dynamic facial stimuli displaying disgust, happiness or neutral expression were examined by means of extended fMRI-constrained source analyses. Behavioral data neither revealed higher recognition accuracy nor higher arousal rates for dynamic as compared to static emotional stimuli, as hypothesized in the introduction. However, neural correlates suggest specific processing characteristics for the different emotional categories, and they provide new insights into the spatio-temporal processing of static and dynamic emotional facial expressions.

In line with our hypotheses, signal space analyses of ERP data showed a modulation of the LPP (Late Posterior Positivity) in both emotional categories including happiness and disgust. The perception of facial expressions of disgust evoked a relative negativity (EPN) at posterior-lateral electrode-sites, descriptively, which, however, did not survive FDR-correction for multiple comparisons. We also expected an emotional modulation of the N170 for static emotional compared to neutral facial expressions, which neither reached statistical significance. In general, voltage topographies suggest that the processing of dynamic as compared to static stimuli yielded a sustained LPP over more widespread electrode sites.

We expected to find a network of posteriorly activated regions for static facial expressions and a more widespread network of both posterior and anteriorly activated brain regions for dynamic facial expression.

FMRI constrained source analysis based on a region of interest (ROI) approach yielded distinct spatio-temporal processing characteristics of emotional faces for the static and dynamic modality. The processing of static facial expressions revealed enhanced source activation in fusiform gyrus for happy and disgust compared to neutrality supporting our hypothesis of posteriorly activated brain regions. The latter area of static emotion processing has not been shown in our previous fMRI study [27] and hence, enlarge the previously described network of emotion processing in static facial expressions.

Furthermore, our results show the time course of dynamic facial expression processing: A more-widespread network (compared to static modality) of early activations in posterior brain areas (i.e. fusiform gyrus (FUG), tuber activation, also cuneus) and in anterior brain areas (i.e. inferior frontal gyrus (IFG), medial frontal gyrus (MFG)) between 200 and 300 ms and ventral medial frontal gyrus (MFG) between 300 and 400 ms has been shown for dynamic facial expressions. In addition, the present source analysis approach revealed the repeated activation of different sources (e.g. medial frontal gyrus and superior frontal gyrus) in the dynamic modality and therefore contributes to our understanding of spatio-temporal processing of both dynamic and static emotional face processing.

There is evidence that advanced methodological approaches, such as the applied source analyses of the present study using prior information revealed by an fMRI-study by using the exact same experimental setup (Trautmann et al., 2009), should improve our understanding of the dynamics of the neural processing of both static and dynamic facial expression processing. It was shown that *a priori* set constraints as for example revealed from locations of fMRI activation foci of previous studies with the same experimental setup, will improve the explanation [23]–[25] and objectivity [45] of source models applied to respective EEG- or MEG-data. Furthermore, the complementary use of both EEG or MEG and functional imaging approaches might enhance the validity of the results as there are neural generator activities, for which the one or the other method is potentially insensitive [24], [26]. This line of argumentation is corroborated by the present data.

### Spatio-temporal Dynamics of Static and Dynamic Facial Expression Processing in Signal Space

In contrast to our hypotheses, only the LPP, but not the N170 and the EPN, component was significantly modulated by emotional category for static emotional faces.

We did not find a significant emotional modulation of the N170 over lateral occipito-temporal regions for both happiness and disgust as compared to neutral facial expressions. The majority of previous studies with healthy participants and patients with prosopagnosia have reported no emotional modulation of this component (for review, see [2], [4], [51], [52]). Latter studies supported the “face perception model” by Bruce and Young [53], which proposes a rather sequential processing of structural and emotional facial characteristics than a parallel processing.

The EPN between 250 and 350 ms was mainly enhanced for disgust at right posterior electrode sites descriptively, but did not survive FDR-correction, which was in contrast to our hypotheses. Previous studies have shown an enhanced EPN either for both positive and negatively valenced emotional expressions [11], [12], or for static angry facial expression [10], for threatening emotional scenes [21], or even for disgusted emotional expressions [9]. Authors of previous studies have interpreted their data in terms of automaticity of processing and/or of a “tagging” of negatively valenced emotional facial expressions, which might possibly be related to reentrant projections to the amygdala [10], [54]. The lack of statistical significance of both the N170 and EPN modulation in the present study might be due to conservative data handling and does not generally rule out an emotional modulation of those components. Further research seems to be necessary to isolate the relevant factors modulating a potential emotional impact on the N170 and EPN component (e.g., individual emotional processing style, specific properties of experimental setup, etc.).

Enhanced LPP was modulated by static facial expressions of disgust over medial parietal regions for several hundreds of milliseconds when FDR-correction for multiple comparisons was applied. In a previous EEG study by Canli and colleagues [42], this sustained positivity was enhanced for up to six seconds. In further previous EEG studies, the LPP has been related to an increased arousal conveyed by emotional faces, which hence, captured attention, maintained a sustained and continuous evaluation of emotional faces, and facilitated a possible transfer to working memory [11], [36].

For the dynamic stimulus modality, there was an emotion-related effect of the LPP for both disgust and happiness, which survived FDR-correction and is furthermore in line with a previous EEG study by Recio et al. [12] also investigating dynamic emotion perception. The reason for finding only an LPP effect and no effects of earlier components for the dynamic modality such as the EPN shown by Recio and colleagues can be explained the nature of the presently used dynamic stimuli. While Recio and and colleagues [12] applied dynamic emotional stimuli, which involved the development of emotional facial expressions from a frontal perspective based on three consecutively presented static stimuli suggesting motion-like expression development, the involved dynamic stimuli of the present approach consisted of realistic continuous video sequences. Faces were initially oriented to the left or right side showing neutral expression before they turned to the front and started their emotional expression simulating a natural social situation. They evoked early potentials only at the beginning of each video (e.g., N170), but not in the middle of it when emotional expressions started developing. The LPP for dynamic compared to static faces on a descriptive level, however, involved a larger amount of electrode positions and was sustained over several hundred milliseconds. This finding possibly reflects the time course of the neural processing of the structural changes of facial features from neutral to the maximum emotional expression, its continuous monitoring and updating of changing facial information and possibly the pre-attentive and/or attentive emotional categorization of the stimuli. This interpretation is in line with the work of Recio and colleagues and others [10], [12], [36]. In the next section, spatio-temporal dynamics of this sustained activation will be discussed on the basis of the respective source analyses data.

### Spatio-temporal Dynamics of Static and Dynamic Facial Expression Processing in Source Space

FMRI constrained source analysis of static happy and disgusted compared to neutral stimuli indicated an engagement of the right fusiform gyrus (FUG), which is a part of the fusiform face area, in the later LPP time window (600–800 ms, Figs. 4 A and B). Thus, enhanced source activation might be related to increased LPP amplitudes for disgust compared to neutral facial expressions over centro-parietal regions. Especially source activations of inferior temporal regions, like the FuG, might reflect a re-entry pattern of information [55] to perception- and/or object-related brain regions.

The corresponding source analysis of dynamic stimulus processing indicated spatio-temporal activation in a widespread network of anterior (e.g., lateral inferior frontal gyrus, medial frontal gyrus, superior frontal gyrus, ventromedial prefrontal cortex), and posterior regions (FUG, tuber, and cuneus, see also Figs. 4 C and D) between 200 and 800 ms after onset of dynamic facial expressions. Dynamic facial expression processing seems to continuously recruit regions, which are discussed to be involved in enhanced processing of dynamically changing structural characteristics of the face (e.g., FuG, [56], [57]), in potentially mirroring motor-related features of face movement (e.g., lateral IFG, and dorsal fronto-medial regions, see also [58]–[60]), and in more deeply evaluating and processing the perceived facial expressions (e.g., inferior frontal and ventro-medial frontal areas, [61], [62]). Especially activation of ventromedial frontal areas during the perception of happy, and hence, positively valenced, dynamic facial expressions might furthermore be related to reward processing [62].

Summarizing, spatio-temporal source space analyses of the present data suggest that static facial emotion-related stimuli trigger late perceptual and/or object-related evaluation processing during the processing of both disgust and happiness.

Dynamic emotion-related stimuli trigger a seemingly homogeneous sustained activation (LPP), which is indeed characterized by alternating posterior and anterior source dynamics, partially acting in a sequential, but also in a parallel way reflecting complex neural network communication and higher order evaluaton processes.

Because of the exploratory approach of the present study, we suggest that the reported temporo-spatial time course of emotion perception (1) should be used for hypotheses building in future investigations of emotion processing, (2) needs replication or falsification in respective future studies, and (3) should also motivate the calculation of different source model approaches (e.g., LAURA, LORETA, Beamforming etc.) with similar experimental designs.

### Behavioral Ratings of Static and Dynamic Facial Expressions

Spanish participants showed an average recognition accuracy of 95.4% for both static and dynamic facial expressions similar to the behavioral rating results displayed by German individuals over all emotional categories, which supports the common notion that facial expressions are universal and can be recognized appropriately and precisely even across different European countries [63], [64].

Contrary to hypotheses presented in the introduction and unlike results presented in our previous fMRI study [27], dynamic stimuli neither yielded significantly better recognition accuracy, nor significantly higher arousal rates compared to static stimuli. Especially higher arousal rates for static compared to dynamic stimuli are in contrast to previous studies [65], [66], reporting higher arousal ratings for dynamic compared to static facial expressions. One might argue that cultural differences of the outer appearance of participants (Spanish, dark skin color and dark/black hair) and actors (light skin color and brown or blonde hair) contributed to this effect. However, cultural differences were not a focus in the present study. Participants noticed, however, predominantly for dynamic faces, the different outer appearance of displayed facial expressions, e.g., lighter skin color and hair. They communicated to the investigator after the categorization session that the outer appearance sometimes appeared unfamiliar to them. This might have attenuated the arousal ratings especially for the dynamic emotional stimuli potentially neutralizing the expected difference effect. Ekman and colleagues (Ekman u. a., 1987) reported similar results and showed that assessing faces from different cultures can result in lower arousal rates for foreign facial expressions because of less experience with the outer appearance and the interpretation of facial expressions of other cultures. An interesting future research question to investigate might be, to what extend individuals need to be familiar with faces of different European countries in order to be able to take advantage of realistic features such as natural movement within a face.

### Limitations of the Study

Because of the known gender differences in emotion perception, we only included female participants to increase homogeneity of the sample. This represents, however, a limitation of the present study because it limits the interpretation of results to the male population. For our exploratory fMRI constrained source analysis approach and regarding the methodological framework for the combination of EEG and fMRI by Hopfinger and colleagues [23], it was important, though, that gender was the same between the present EEG and our previous fMRI study. Future dynamic and static emotion perception designs applying fMRI constrained source analysis should, hence, investigate gender differences in detail to enhance generalizability of present and future results.

Another important aspect is the fact that static and dynamic modalities can not be compared directly in a spatio-temporal way. This is an inherent problem caused by the different temporal characteristics of the stimuli. While static expressions showed the maximum emotional expression with stimulus onset and, therfore, incorporated an immediate social presence, dynamic stimuli included a turn of a neutral face to the front before the actual emotional expression developed from neutral to emotional. The reason for using this experimental setup was to introduce a social and natural context: The head turning towards an observer is a highly salient signal and provides important features of a realistic social scenario. However, a previous study by Recio and colleagues [12] showed that dynamic stimuli morphing from neutral to emotional without the turn of the face were also able to elicit emotionally modulated EPN and LPP. The major difference between their stimuli and the stimulus material of the present study was that their stimuli were virtually constructed by a video software, while our stimuli consisted of real actors sitting in front of a video camera and presenting emotional expressions. We assume that both stimulus sets provide advantages: While Recio and co-workerś stimuli were highly controlled for timing, our stimuli had the strong advantage of real-life changing facial expression dynamics potentially enhancing ecological validity.

For future studies it would be interesting to investigate (1) the differences between artificially morphed dynamic stimulus material compared to real moving facial expressions both in relation to ratings of naturalness and the respective neural correlates, and (2) to compare dynamic emotional face processing of real moving facial expressions with and without the turn to the front to better disentangle potential social relevance of particular stimulus features.

### Final Conclusions

Emotions in human’s everyday social interactions are dynamic in nature and need to be monitored continuously and intensely. The analysis of static and dynamic facial expressions is inherently different regarding the different structural features and temporal characteristics. Several authors used complex video stimuli to examine brain activity in socially relevant contexts and discuss their results in relation to potentially larger ecological validity of dynamic and more natural stimuli [12], [27], [67]–[69]. Dynamic emotional expressions might provide a more realistic basis, and they have further been shown to enrich emotional perception of facial expressions [65]. During social communication in everyday life, individuals are accustomed to watching dynamic expressions evolving from one facial expression (e.g., neutral) to the next facial expression (e.g., happiness or disgust). Therefore, in daily social interactions they are more experienced in watching and analyzing dynamically changing facial expressions [32], which potentially trigger more widespread neural resources [27] for a sufficient analysis of facial expressions given their contextual complexity. This might also be reflected in the enhanced and prolonged LPP for dynamic faces, which can be explained by alternating perceptually and conceptually related regional brain activations indicated by the respective source analyses.

Summarizing, fMRI constrained source analysis revealed different processing dynamics of empathic perception of static and dynamic facial expressions at different locations in the brain. The present results emphasize (1) the importance of studying dynamic emotional face processing in future studies in more detail, and (2) different spatio-temporal and topographical characteristics of dynamic and static facial expression processing.
